# WMJ-S-001, a Novel Aliphatic Hydroxamate-Based Compound, Suppresses Lymphangiogenesis Through p38mapk-p53-survivin Signaling Cascade

**DOI:** 10.3389/fonc.2019.01188

**Published:** 2019-11-06

**Authors:** Shiu-Wen Huang, Hung-Yu Yang, Wei-Jan Huang, Wei-Chuan Chen, Meng-Chieh Yu, Shih-Wei Wang, Ya-Fen Hsu, Ming-Jen Hsu

**Affiliations:** ^1^Department of Medical Research, Taipei Medical University Hospital, Taipei, Taiwan; ^2^Department of Pharmacology, School of Medicine, College of Medicine, Taipei Medical University, Taipei, Taiwan; ^3^Division of Cardiovascular Medicine, Department of Internal Medicine, Taipei Medical University-Wan Fang Hospital, Taipei, Taiwan; ^4^Department of Internal Medicine, School of Medicine, College of Medicine, Taipei Medical University, Taipei, Taiwan; ^5^Graduate Institute of Pharmacognosy, Taipei Medical University, Taipei, Taiwan; ^6^Graduate Institute of Medical Sciences, College of Medicine, Taipei Medical University, Taipei, Taiwan; ^7^Department of Medicine, Mackay Medical College, New Taipei City, Taiwan; ^8^Graduate Institute of Natural Products, College of Pharmacy, Kaohsiung Medical University, Kaohsiung, Taiwan; ^9^Division of General Surgery, Department of Surgery, Landseed Hospital, Taoyuan, Taiwan

**Keywords:** hydroxamate, lymphangiogenesis, lymphatic endothelial cells (LECs), p53, p38, survivin

## Abstract

**Background and purpose:** Angiogenesis and lymphangiogenesis are major routes for metastatic spread of tumor cells. It thus represent the rational targets for therapeutic intervention of cancer. Recently, we showed that a novel aliphatic hydroxamate-based compound, WMJ-S-001, exhibits anti-angiogenic, anti-inflammatory and anti-tumor properties. However, whether WMJ-S-001 is capable of suppressing lymphangiogenesis remains unclear. We are thus interested in exploring WMJ-S-001's anti-lymphangiogenic mechanisms in lymphatic endothelial cell (LECs).

**Experimental approach:** WMJ-S-001's effects on LEC proliferation, migration and invasion, as well as signaling molecules activation were analyzed by immunoblotting, flow-cytometry, MTT, BrdU, migration and invasion assays. We performed tube formation assay to examine WMJ-S-001's *ex vivo* anti-lymphangiogenic effects.

**Key results:** WMJ-S-001 inhibited serum-induced cell proliferation, migration, invasion in murine LECs (SV-LECs). WMJ-S-001 reduced the mRNA and protein levels of survivin. Survivin siRNA significantly suppressed serum-induced SV-LEC invasion. WMJ-S-001 induced p53 phosphorylation and increased its reporter activities. In addition, WMJ-S-001 increased p53 binding to the promoter region of survivin, while Sp1 binding to the region was decreased. WMJ-S-001 induced p38 mitogen-activated protein kinase (p38MAPK) activation. p38MPAK signaling blockade significantly inhibited p53 phosphorylation and restored survivin reduction in WMJ-S-001-stimulated SV-LCEs. Furthermore, WMJ-S-001 induced survivin reduction and inhibited cell proliferation, invasion and tube formation of primary human LECs.

**Conclusions and Implications:** These observations indicate that WMJ-S-001 may suppress lymphatic endothelial remodeling and reduce lymphangiogenesis through p38MAPK-p53-survivin signaling. It also suggests that WMJ-S-001 is a potential lead compound in developing novel agents for the treatment of lymphangiogenesis-associated diseases and cancer.

## Introduction

Lymphangiogenesis, the formation of new lymphatic vessels, occurs primarily in many physiological processes such as embryonic development, tissue repair and resolution of inflammatory reactions. It also contributes to a variety of pathological events including lymphedema, inflammatory diseases, and tumor metastasis (1). Metastatic spread of tumor cells is the major cause of morbidity and mortality and responsible for ~90% of cancer-related deaths (2). A variety of mechanisms such as seeding of body cavities, local tissue invasion, invasion into lymphatics and hematogenous spread are involved in metastatic tumor spread. Although both blood and lymphatic systems contribute to tumor progression and metastasis, the dissemination of tumor cells via lymphatic vasculature is the most common route for most carcinomas (3, 4). Increased number of tumor-associated lymphatic vessels has been shown closely correlated with metastasis and poor clinical outcome (5).

Lymphangiogenesis, similar to angiogenesis, is tightly regulated by lymphangiogenic factors and its cognate receptors (6). The member of the vascular endothelial growth factor (VEGF) family, VEGF-C, is currently the best-characterized lymphangiogenic factor. VEGF-C's lymphangiogenic effects is primarily mediated by VEGFR-3 (also known as flt-4). The expression of VEGFR-3 is largely restricted to the lymphatic endothelial cells (LECs) in normal adult tissues (7, 8). It is reported in experimental xenograft models that VEGF-C stimulates tumor lymphangiogenesis, as well as lymph node metastasis (9, 10). In addition, VEGF-C overexpression in tumor tissues significantly correlates with accelerated tumor progression and poor clinical outcome (11). Knocking down VEGF-C expression with siRNA significantly prevented lymphangiogenesis and enhanced chemo-sensitivity in breast cancer cells (12). Therefore, VEGF-C-associated lymphangiogenesis has emerged as a key prognostic marker and represents a promising therapeutic target for cancer intervention (13). To interfere with VEGF-C-VEGFR-3 signaling, a variety of strategies has been reported currently. These includes neutralizing antibodies or peptides that antagonize VEGFR-3 signaling, receptor traps or monoclonal antibodies targeting VEGF-C and small molecule receptor tyrosine kinase inhibitors of VEGFR-3 (14).

The smallest member of the inhibitor of apoptosis protein (IAP) family, survivin, is rarely detected in most terminally differentiated adult tissues with notable exceptions of vascular endothelial or hematopoietic cells (15). However, a high survivin level is found in most common human cancers. Highly expressed survivin positively correlates with tumor progression and poor prognosis (16–18). Survivin not only suppresses cell death, but also participates in a variety of cellular events. These include cell migration, cell cycle progression (16), and angiogenesis, which may promote metastatic spread of tumor cells (17). Cai et al. (19) reported that survivin level is associated with VEGF-C level and the presence of lymphatic invasion in breast cancer. However, the association between endothelial survivin and lymphangiogenesis remains incompletely understood. It is likely that modulating survivin level may provide another means of regulating tumor lymphangiogenesis. Survivin expression is regulated primarily at the transcriptional level. Survivin is up-regulated by transcription factors such as signal transducer and activator of transcription 3 (STAT3) (20), specificity protein 1 (Sp1) (21) or hypoxia-inducible factor-1α (HIF-1α). However, p53, a tumor suppressor, may cause survivin reduction (22, 23). Pharmacological targeting of the p53-survivin cascade may be a potential therapeutic strategy in not only causing tumor cell death, but also suppressing lymphangiogenesis and tumor progression.

Hydroxamate, a key pharmacophore, has attracted considerable attention in drug development field due to its diverse pharmacological properties (24). Growing evidence demonstrates the potential use of hydroxamate derivatives as anti-tumor (20, 25), anti-inflammatory (26), or anti-infectious (27) agents. Recently, we synthesized and showed that a novel aliphatic hydroxamate-based compound WMJ-S-001 exhibits anti-tumor (22), anti-inflammatory (28) and anti-angiogenic (20) properties. Given its potential as lead compound for drug discovery, we aimed to investigate WMJ-S-001's anti-lymphangiogenic effects and its underlying mechanisms in LECs.

## Materials and Methods

### Reagents

All cell culture reagents including fetal bovine serum (FBS), TrypLE™, DMEM medium and transfection reagent, Turbofect^TM^ were from Invitrogen (Carlsbad, CA, U.S.A.). All chemicals including toluidine blue O and 3-[4, 5-dimethylthiazol-2-yl]-2, 5-diphenyltetrazolium bromide (MTT) were from Sigma-Aldrich (St Louis, MO, U.S.A.). Ribociclib was from MedChemExpress (Monmouth Junction, NJ, U.S.A.). Antibodies against PARP, caspase 3 active form, survivin, ERK1/2 and ERK1/2 phosphorylated at Thr 202/Tyr 204, p38MAPK, p38MAPK phosphorylated at Thr180/Tyr182, p53 phosphorylated at Ser15 and p53 acetylated at Lys379 were from Cell Signaling (Danvers, MA, U.S.A.). Antibodies against α-tubulin and GAPDH, as well as anti-rabbit and anti-mouse IgG conjugated horseradish peroxidase antibodies were from GeneTex Inc (Irvine, CA, U.S.A.). Antibody against Sp1 and normal IgG were from Santa Cruz Biotechnology (Santa Cruz, CA, USA). Immobilon Western Chemiluminescent HRP Substrate was from Millipore (Billerica, MA, USA). All materials for western analysis were from Bio-Rad (Hercules, CA, U.S.A.). Cell Proliferation ELISA, BrdU assay kit was from Roche (Indianapolis, IN, USA). PG13-luciferase construct (p53-luc) with p53 binding sites (Addgene plasmid #16642) and p21/WAF1 promoter luciferase construct (p21 pro-luc, Addgene plasmid #16451) as described previously (29) were kindly provided by Dr. Bert Vogelstein. A Dual-Glo luciferase assay system and renilla-luciferase construct were from Promega (Madison, WI, U.S.A.).

### Synthesis of WMJ-S-001

WMJ-J compounds were synthesized as described previously (28).

### Cell Culture

The murine LEC line SV-LEC was kindly provided by Dr. J.S. Alexander (Shreveport, LA). SV-LECs were cultured as previously described (30). Primary human lymphatic endothelial cells (HLEC, C-12217), MV2 basal and growth medium were purchased from PromoCell (Heidelberg, Germany). HLECs were maintained in MV2 growth medium in a humidified 37°C incubator. The MV2 growth medium contains growth supplements including 5% fetal calf serum, 5 ng/ml epidermal growth factor, 10 ng/ml basic fibroblast growth factor, 20 ng/ml insulin-like growth factor, 0.5 ng/ml vascular endothelial growth factor, 1 μg/ml ascorbic acid, 0.2 μg/ml hydrocortisone.

### MTT Assay

We used the colorimetric MTT assay to determine cell viability as described previously (28).

### Cell Proliferation Assay (BrdU Incorporation Assay)

Human lymphatic endothelial cells (HLECs) (2 × 10^4^ per well) seeded in 48-well tissue culture plates were starved in MV2 basal medium in the absence of growth supplements for 24 h. After starvation, cells were incubated in MV2 growth medium containing growth supplements with or without WMJ-S-001 at indicated concentrations for another 24 h. A Cell Proliferation ELISA, BrdU (colorimetric) kit (Roche) based on the colorimetric detection of the incorporation of BrdU was used to determine cell proliferation following the manufacturer's instructions.

### Flowcytometry

SV-LCEs were treated with indicated concentrations of WMJ-S-001 for 24 h. Cells were harvested and fixed in 70% ethanol at 0°C for another 24 h. After fixation and washed with phosphate-citric acid buffer, cells were stained in the dark for 30 min with staining buffer [(0.1% Triton X-100, 100 μg/ml RNase A and 25 μg/ml propidium iodide (PI)]. The FACScan and Cellquest program (BD Biosciences, San Jose, CA, U.S.A.) were used to perform flow-cytometric analysis. The percentage of PI-stained cells in the G0/G1, S, G2/M, or subG1 (Apoptosis, Apo) region was analyzed using the FCS Express (De Novo Software, Glendale, CA, U.S.A) or ModFit (BD Biosciences, San Jose, CA, U.S.A.) program.

### Western Analysis

After treatment, cells were harvested in lysis buffer [0.5% NP-40, 140 mM NaCl, 10 mM Tris (pH 7.0), 0.05 mM pepstatin A, 2 mM PMSF and 0.2 mM leupeptin]. Cell lysate with equal amounts of protein were subjected to SDS-PAGE and transferred onto a NC membrane (Pall Corporation, Washington, NY, U.S.A.). After transfer, membrane was incubated with 5% non-fat milk-containing blocking buffer for 1 h. Specific primary antibodies and horseradish peroxidase-conjugated secondary antibodies were used to recognize target proteins. Enhanced chemiluminescence was used to detect immunoreactivity according to manufacturer's instructions. To obtain the quantitative data, a computing densitometer with a scientific imaging system (Biospectrum AC System, UVP) was used.

### Cell Transfection

SV-LECs (7 × 10^4^ cells/well) were transfected with PG13-luciferase (p53-luc) or p21 promoter-luciferase (p21 pro-luc) plus renilla-luciferase for reporter assay or transfected with negative control siRNA (NC) or survivin siRNA for MTT, flowcytometry, immunoblotting and invasion assay using Turbofect transfection reagent (Invitrogen, Carlsbad, CA, U.S.A.) per manufacturer's instructions.

### Reporter Assay

After transfection, SV-LECs were treated with vehicle or WMJ-S-001 for another 24 h. A Dual-Glo luciferase assay system kit (Promega, Madison, WI, U.S.A.) was employed to determine the luciferase reporter activity per manufacturer's instructions. The reporter activity was normalized based on renilla-luciferase activity.

### Survivin Silencing

Target gene silencing in SV-LECs was performed as described previously (31). Negative control scramble siRNA and pre-designed siRNAs targeting the murine *survivin (BIRC5)* were purchased from Sigma-Aldrich (St Louis, MO, U.S.A). The siRNA oligonucleotides were as follows: *survivin* siRNA, 5′-cgauagaggagcauagaa-3′ and negative control scramble siRNA, 5′-gaucauacgugcgaucaga-3′.

### Cell Migration Assay

SV-LECs were seeded in the 12-well tissue culture plates. After growing to confluence, SV-LECS were starved with serum-free DMEM medium for 24 h. Pipette tips were used to create scratch wounds in monolayers of SV-LECs. Cells were washed with PBS, followed by the treatment with WMJ-S-001 at different concentrations in the presence or absence of 10% FBS for another 24 h. Cells were fixed with cold 4% paraformaldehyde and stained with 0.5% toluidine blue. After staining, an *OLYMPUS* Biological Microscope digital camera (Yuan Li Instrument Co., Taipei, Taiwan) was used to take photographs at 40× magnification. Cell migration rate was determined by calculating the migrated cells in the wound area.

### Invasion Assay

We performed cell invasion assays as described previously (20). 0.2% gelatin solution was used to coat the lower face of the filter in the transwell plate (Corning, NY, U.S.A.). The lower chambers were filled with containing 10% FBS-containing DMEM medium (SV-LECs) or growth supplements-containing MV2 medium (HLECs). SV-LECs or HLECs (2 × 10^4^ cells per chamber) were seeded in the upper chambers in the serum-free DMEM medium or MV2 basal medium with or without WMJ-S-001. After 18 h, the non-invaded cells in the upper chamber were removed by gently scraped with a cotton swab. The invaded cells in the lower face of the filter were fixed, stained with toluidine blue (0.5% in 4% paraformaldehyde) and photographed using an optical microscope (Nikon, Japan) at ×40. The number of stained cells that invaded through the filter were counted. We also quantified cell invasion by dissolving the stained cells in 33% acetic acid and measuring the absorbance at 570 nm.

### Reverse-Transcription-Quantitative Real-Time Polymerase Chain Reaction (RT-qPCR)

After treatment as indicated, cells were harvested for isolation of total RNA and complementary DNA (cDNA) synthesis as previously described (31). We used GoTaq qPCR Master Mix (Promega, Madison, WI, U.S.A.) and StepOne Real-Time PCR systems (Applied Biosystems, Grand Island, NY U.S.A.) to perform RT- qPCR. The cycling conditions were as follows: hot-start activation for 2 min at 95°C, followed by 40 cycles of denaturation for 15 s at 95°C, annealing/extension for 60 s at 60°C. The primers used to transcribe survivin and GAPDH are as follows: human survivin forward, 5′-gcctttccttaaaggccatc-3′; human survivin reverse, 5′-aacccttcccagactcca ct-3′; human GAPDH forward, 5′-gtcagtggtggacctgacct-3′; human GAPDH reverse, 5′-aggggtctacatggcaactg-3′; mouse survivin forward, 5′-atcgccaccttcaagaactg-3′; mouse survivin reverse, 5′-tgactgacgggtagtctttgc-3′; mouse GAPDH forward, 5′-ccttcattgacctcaactac-3′; mouse GAPDH reverse, 5′-ggaaggccatgccagtgagc-3′.

### Chromatin Immunoprecipitation (ChIP) Assay

After treatment as indicated, cells were cross-linked with formaldehyde (1%) for 10 min at 37°C. Cross-linking was quenched by adding 1.25 M glycine. After harvesting cells with ice-cold PBS, the cell pellet was resuspended in SDS lysis buffer. Samples were sonicated five times (for 15 s each) and centrifuged (10 min) to collect supernatants. An aliquot of each sample was used as “Input.” The remainder of the soluble chromatin was diluted in ChIP dilution buffer. Immunoprecipitation was performed by adding normal IgG, anti-p53, or anti-Sp1 antibodies plus protein A-magnetic beads (Millipore, Billerica, MA, U.S.A.) with a gentle rotation at 4°C for 18 h. The immune complexes were washed sequentially in the following buffers: low-salt, high-salt, LiCl immune complex washing buffer and Tris-EDTA buffer. After last wash, elution buffer (100 μl each) was added twice to elute the immune complex. The cross-linked chromatin complex was reversed by adding 0.2 M NaCl and heating for 4 h at 65°C. GP^TM^ DNA purification spin columns (Viogene, New Taipei City, Taiwan) were used to purify DNA. Purified DNA was used to perform PCR with PCR Master Mix (Promega, Madison, WI, U.S.A.). To amplify the *survivin* promoter fragment, the following primers were used: forward, 5′-accgcagcagaaggtacaac-3′ and reverse, 5′-agacgactcaaacgcaggat-3′. The cycling conditions were as follows: initial denaturation for 5 min at 95°C, followed by 30-cycles of 30 s at 95°C, 30 s at 56°C and 45 s at 72°C and final extension for another 10 min at 72°C. PCR products were analyzed by agarose gel electrophoresis (1.5%).

### Tube Formation Assay

The basement membrane matrix, matrigel (Becton Dickinson, Mountain View, CA, USA) was used to perform the tube formation assay as previously described (32). Matrigel was polymerized for 30 min at 37°C. HLECs were seeded onto the matrigel in MV2 basal medium with or without WMJ-S-001 at indicated concentrations. After 24 h, cells were photographed using an optical microscope (Nikon, Japan) at ×40.

### Blinding and Randomization

We have different people analyzing data (analyst) and conducting experiments (operator) for blinding. The same cell in every single experiment was used to evaluate the WMJ-S-001's effects vs. the related control. Therefore, formal randomization was not employed.

### Data and Statistical Analysis

In this study, the data and statistical analysis comply with the recommendations on experimental design and analysis in pharmacology (33). Results represented as mean ± standard error of mean (S.E.M) (*n* ≥ 3), where ‘*n*' refers to independent values, and not replicates. Normalization was performed to compare the differences after the treatment to control for unwanted sources of variation and to reveal relevant trends. To reduce the effect of variation from different exposure of immunoblotting, the protein expression levels or the status of protein modification were expressed by normalization that generates control values with no variance (SEM = 0). Such data are not subjected to parametric statistical analysis. SigmaPlot 10 (Build 10.0.0.54; Systat Software, San Jose, CA, U.S.A.) was used to perform statistical analysis. Statistical comparisons between two groups were evaluated by the Mann–Whitney test for non-parametric analysis or unpaired Student's *t*-test for parametric analysis. Kruskal-Wallis test followed by Dunn's multiple comparison for non-parametric analysis or one-way analysis of variance (ANOVA) with Tukey's *post-hoc* test for parametric analysis was used to evaluate the statistical comparisons between more than two groups. A *P*-value smaller than 0.05 was defined as statistically significant.

## Results

### WMJ-S-001 Inhibits SV-LEC Proliferation and Causes Apoptosis

**Figure 1:**
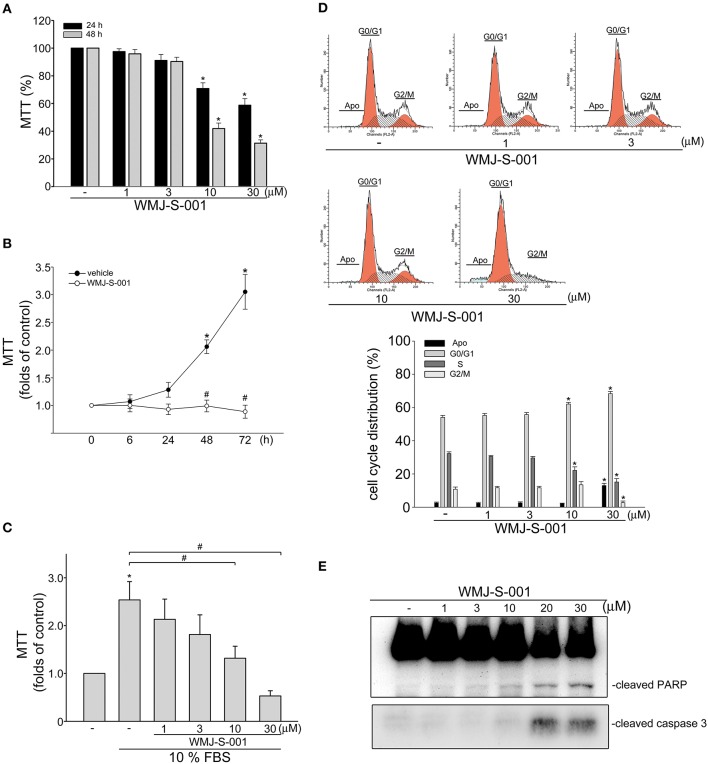
WMJ-S-001 inhibited cell proliferation and induced apoptosis in SV-LECs. **(A)** SV-LECs were treated with indicated concentrations of WMJ-S-001 for 24 or 48 h. Cell viability was determined by MTT assay. Each column represents the mean ± S.E.M. of eight independent experiments performed in duplicate (*p < 0.05, compared with the control group). **(B)** SV-LECs were starved in serum-free DMEM medium for 24 h. After starvation, cells were treated with vehicle or WMJ-S-001 (10 μM) in the presence of 10% FBS for indicated periods. Cell viability was determined by MTT assay. Each column represents the mean ± SEM of five independent experiments performed in duplicate (*p < 0.05, compared with the control group at the time 0; #*p* < 0.05, compared with the vehicle-treated control group at the same time point). **(C)** After starvation as described in **(B)**, cells were treated with vehicle or indicated concentrations of WMJ-S-001 in the presence of 10% FBS for 48 h. Cell viability was determined by MTT assay. Each column represents the mean ± S.E.M. of six independent experiments performed in duplicate (*p < 0.05, compared with the control group; #*p* < 0.05, compared with the control group in the presence of 10% FBS). **(D)** Cells were treated with vehicle or WMJ-S-001 at indicated concentrations for 24 h. The percentage of cells in subG1, G0/G1, S, and G2/M phases was then analyzed by flow-cytometric analysis with PI staining. Each column represents the mean ± S.E.M. of five independent experiments. (*p < 0.05, compared with the control group) **(E)** Cells were treated as in **(D)**, the extent of cleavage caspase 3 and PARP were then determined by immunoblotting. Results shown are representative of four independent experiments.

It is still difficult to isolate and propagate LECs from different organs (7, 34, 35). This limits the studies on lymphangiogenesis or signaling mechanisms in LECs. In this study, we selected a SV40 large T-expressed immortalized murine LEC line (SV-LEC), which retain their “lymphatic” endothelial characteristics after repeated passages (30, 36), to overcome these limitations. A MTT assay was used to examine whether WMJ-S-001, a novel aliphatic hydroxamate-based compound, affects SV-LEC viability. As shown in Figure 1A, WMJ-S-001 reduced SV-LEC viability in the concentration- and time-dependent manners. We next determined whether WMJ-S-001's inhibitory actions on the cell viability attributes to its anti-proliferative effects. After starvation with serum-free medium for 24 h, SV-LECs were stimulated with serum (10% FBS) for another 6 to 72 h in the presence or absence of WMJ-S-001 (10 μM). As shown in Figure 1B, WMJ-S-001 significantly reduced serum-induced SV-LEC proliferation. In addition, WMJ-S-001 concentration-dependently inhibited cell proliferation in SV-LECs after 48 h exposure to serum (Figure 1C). We next used flow cytometry with propidium iodide (PI) staining to examine whether WMJ-S-001 alters cell cycle progression or induces apoptosis. Treatment of WMJ-S-001 for 24 h significantly reduced the percentage of PI-stained cells in the S region as compared with the control group (Figure 1D). This effect was accompanied by a concomitant increase in the percentage of PI-stained cells in the G1 region after treatment of WMJ-S-001 (Figure 1D). In addition, WMJ-S-001 at concentrations of 10 μM or lower did not significantly cause apoptosis (sub-G1, apoptotic region). However, WMJ-S-001 at 30 μM significantly induced cell apoptosis in SV-LECs (Figure 1D). We next examined whether WMJ-S-001 activates caspase3, an apoptosis marker. WMJ-S-001 at concentrations higher than 10 μM (20 and 30 μM) markedly increased the cleaved (active) form of caspase 3 and its substrate, PARP (Figure 1E). It suggests that inhibiting LEC proliferation and causing apoptosis may contribute to WMJ-S-001's anti-lymphangiogenic effects.

### WMJ-S-001 Inhibits Serum-Induced LEC Migration and Invasion

**Figure 2:**
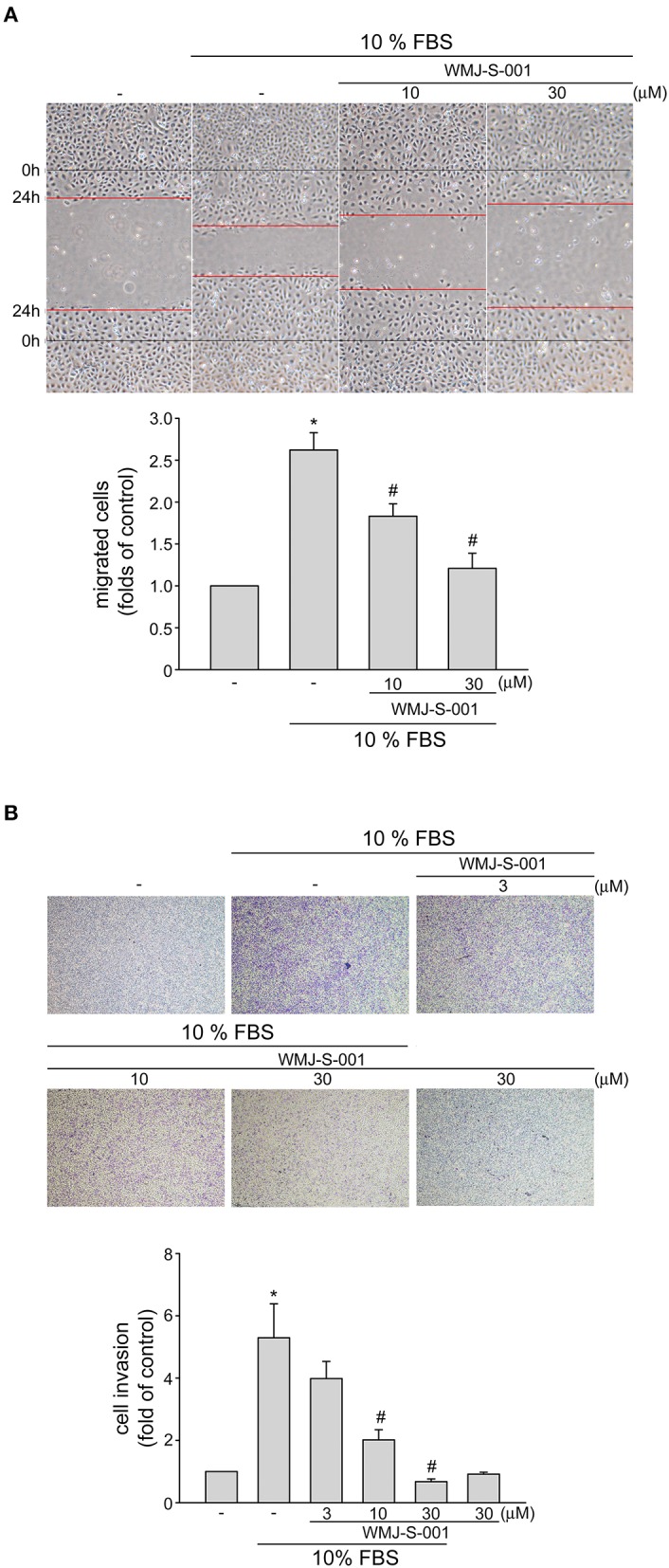
WMJ-S-001 inhibited SV-LEC migration and invasion in SV-LECs. **(A)** SV-LECs were starved in serum-free DMEM medium for 24 h. After starvation, cells were scratched and treated with vehicle or WMJ-S-001 at indicated concentrations in the presence of serum (10% FBS) for another 24 h. The rate of cell migration was determined as described in the section Materials and Methods. Each column represents the mean ± S.E.M. of five independent experiments (*p < 0.05, compared with the control group; #*p* < 0.05, compared with the group treated with serum alone). **(B)** A total of 10^4^ SV-LECs were seeded in the top gelatin-coated chamber and treated with vehicle or indicated concentrations of WMJ-S-001 using serum (10% FBS) as chemo-attractant. After 16 h, the SV-LECs that invaded through the gelatin-coated membrane were stained and quantified as described in the section Materials and Methods. Each column represents the mean ± S.E.M. of six independent experiments (*p < 0.05, compared with the control group; #*p* < 0.05, compared with the group treated with serum alone).

We next examined whether WMJ-S-001 alters cell motility, a pivotal step in lymphangiogenesis, in SV-LECs after serum (10% FBS) exposure. WMJ-S-001 significantly inhibited serum-induced LEC migration as determined by wound-healing migration assay (Figure 2A). We also used transwell invasion assay to examine WMJ-S-001's effects on serum-induced cell invasion. As shown in Figure 2B, WMJ-S-001 at 10 and 30 μM significantly reduced the number of invading cell penetrating the gelatin-coated transwell filter barrier, using serum (10% FBS) as the chemoattractant. These observations indicate that WMJ-S-001 is capable of inhibiting LEC migration and invasion.

### Surivin Reduction Contributes to WMJ-S-001's Inhibitory Effects on SV-LEC Invasion

**Figure 3:**
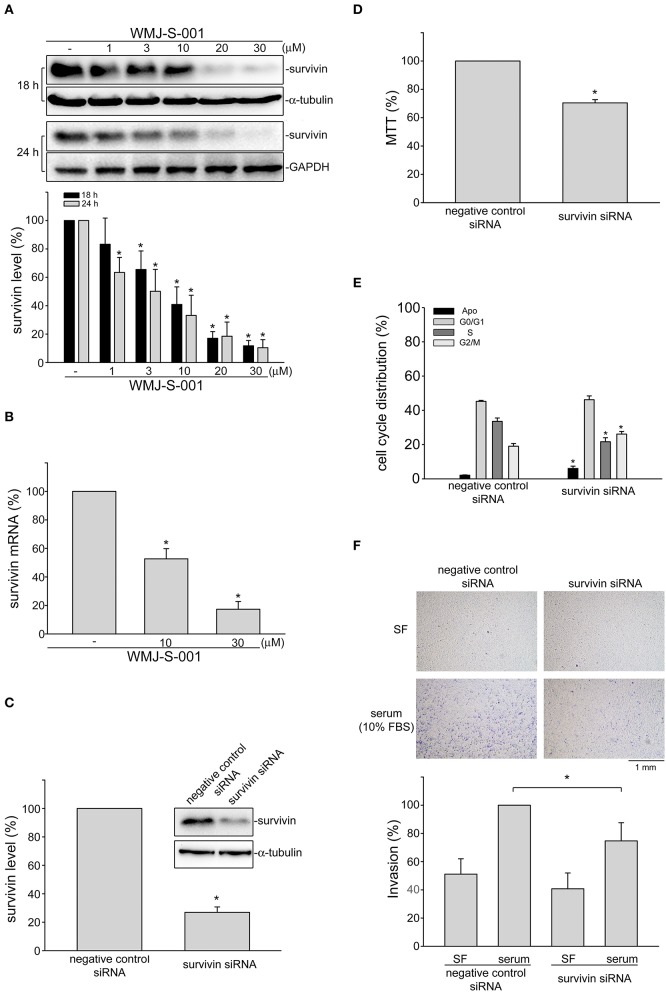
WMJ-S-001 induced survivin reduction in SV-LECs. **(A)** Cells were treated with WMJ-S-001 at indicated concentrations for 18 or 24 h. The extent of survivin was determined by immunoblotting. Each column represents the mean ± S.E.M. of five independent experiments (*p < 0.05, compared with the control group). **(B)** Cells were treated with WMJ-S-001 at indicated concentrations for 6 h. The extent of survivin mRNA was determined by Q-RT-PCR as described in the section Materials and Methods. Each column represents the mean ± S.E.M. of five independent experiments (*p < 0.05, compared with the control group). **(C)** Cells were transfected with negative control siRNA or survivin siRNA for 48 h. After transfection, cells were harvested and the extent of survivin level was determined by immunoblotting. Each column represents the mean ± S.E.M. of five independent experiments (*p < 0.05, compared with the negative control siRNA-transfected group). **(D)** After transfection as described in **(C)**, cell viability was determined by MTT assay. Each column represents the mean ± S.E.M. of five independent experiments performed in duplicate (*p < 0.05, compared with the negative control siRNA-transfected group). **(E)** After transfection as described in **(C)**, flow-cytometric analysis was used to determine cell cycle distribution. Each column represents the mean ± SEM of five independent experiments (*p < 0.05, compared with the negative control siRNA-transfected group). **(F)** After transfection as described in **(C)**, cells were harvested for cell invasion assay as described in the section Materials and Methods. Each column represents the mean ± S.E.M. of five independent experiments (*p < 0.05, compared with the negative control siRNA-transfected group in the presence of serum). SF, serum free.

Growing evidence has demonstrated that suvivin not only regulates mitosis and apoptosis, but also plays a critical role in angiogenesis (37, 38). However, whether endothelial survivin participates in lymphangiogenesis remains unclear. We thus determined whether WMJ-S-001 alters survivin level in SV-LECs. Results from immunoblotting analysis demonstrated that survivin exhibits high expression levels in SV-LECs (Figure 3A). However, treatment of cells with WMJ-S-001 for 18 or 24 h concentration-dependently caused survivin reduction (Figure 3A). We also determined whether WMJ-S-001 affects survivin mRNA level in SV-LECs. As shown in Figure 3B, WMJ-S-001 at 10 or 30 μM significantly reduced survivin mRNA expression. It indicates that WMJ-S-001 may reduce survivin expression at the transcriptional level. A survivin siRNA oligonucleotide was employed to determine whether survivin reduction decreases cell viability in SV-LECs. Survivin siRNA significantly reduced the basal level of survivin (Figure 3C) and decreased cell viability (Figure 3D) in SV-LECs. Results from flow-cytometric analysis further demonstrated that survivin reduction mimics the enhancing effects of WMJ-S-001 in reducing the percentage of PI-stained cells in the S region. Survivin siRNA also caused G2/M cell cycle arrest and apoptosis in SV-LECs (Figure 3E). We examined whether survivin reduction affects SV-LEC motility. As shown in Figure 3F, survivin siRNA mimicked the WMJ-S-001's effects in reducing the number of invading cell penetrating the gelatin-coated transwell filter barrier, using serum as the chemoattractant. In contrast, negative control siRNA was without effects (Figure 3F). These observations suggest that survivin reduction may contribute to WMJ-S-001's inhibitory effects on SV-LEC motility and subsequent lymphangiogenesis.

### p38MAPK Mediates WMJ-S-001-Induced p53 Activation and Survivin Reduction in SV-LECs

**Figure 4:**
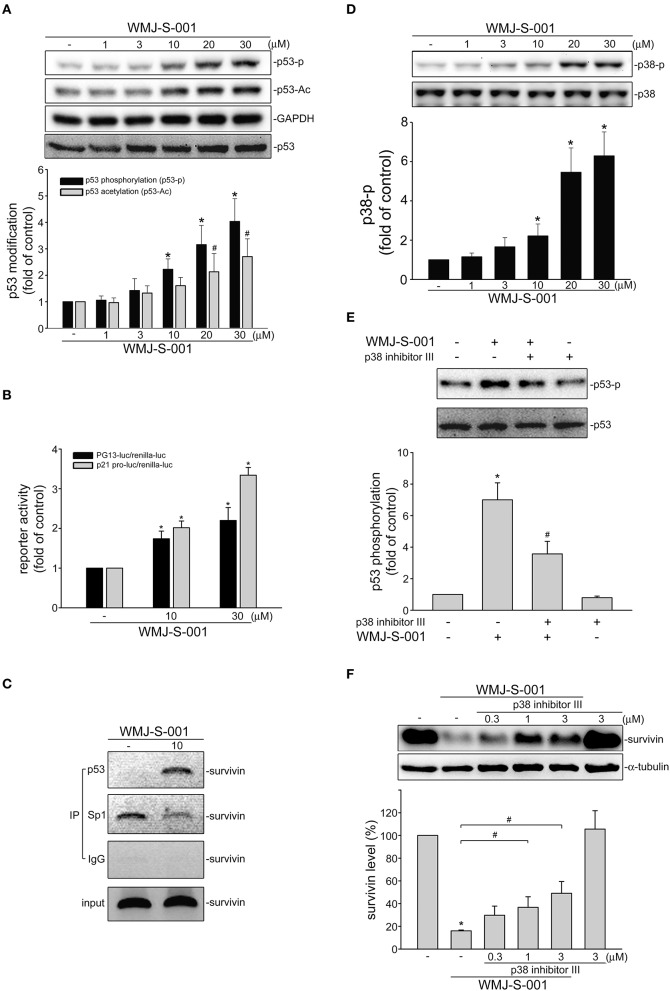
WMJ-S-001 induced p38MAPK-p53 activation in SV-LECs. **(A)** Cells were treated with vehicle or WMJ-S-001 at indicated concentrations for 6 h. The phosphorylation and acetylation status of p53 was determined by immunoblotting. Each column represents the mean ± S.E.M. of five independent experiments (*p < 0.05, compared with the control group). **(B)** Cells were transfected with PG13-luc (p53-luc) or p21 promoter-luc plus renilla-luc for 48 h. After transfection, cells were stimulated with WMJ-S-001 at indicated concentrations for another 24 h. Luciferase activity was then determined. Each column represents the mean ± S.E.M. of six independent experiments (*p < 0.05, compared with the control group). **(C)** Cells were treated with WMJ-S-001 (10 μ M) for 6 h. ChIP assay was then performed as described in the section Materials and Methods. Typical traces representative of three independent experiments with similar results are shown. **(D)** Cells were treated with vehicle or WMJ-S-001 at indicated concentrations for 6 h. The phosphorylation status of p38MAPK was determined by immunoblotting. Each column represents the mean ± S.E.M. of six independent experiments (*p < 0.05, compared with the control group). **(E)** Cells were treated with p38 inhibitor III (1 μM) for 30 min followed by WMJ-S-001 (10 μM) exposure for another 6 h. The phosphorylation status of p53 was determined by immunoblotting. Each column represents the mean ± S.E.M. of five independent experiments (*p < 0.05, compared with the control group; #*p* < 0.05, compared with the group treated with WMJ-S-001 alone). **(F)** Cells were treated with p38 inhibitor III at indicated concentrations for 30 min followed by WMJ-S-001 (20 μM) exposure for another 24 h. The extent of survivin was determined by immunoblotting. Each column represents the mean ± S.E.M. of five independent experiments. (*p < 0.05, compared with the control group; #*p* < 0.05, compared with the group treated with WMJ-S-001 alone).

We investigated the underlying mechanisms of WMJ-S-001 in repressing survivin expression in SV-LECs. Transcription factor p53 participates in various cellular processes such as cell cycle arrest, apoptosis or senescence by regulating diverse target genes (39). It is reported that p53 may suppress survivin expression by counteracting Sp1 binding to the survivin promoter region (40). Therefore, we explored whether WMJ-S-001 induces p53 activation in SV-LECs. Post-translational modifications such as phosphorylation or acetylation play important roles in regulating p53's activity (41). We thus examined WMJ-S-001's effects on p53 phosphorylation and acetylation in SV-LECs. As shown in Figure 4A, 6 h exposure to WMJ-S-001 led to concentration-dependent increases in p53 phosphorylation and acetylation. Results from reporter assay showed that treatment of cells with WMJ-S-001 for 24 h significantly increased PG13-luciferase (p53-luciferase) activity. WMJ-S-001 also increased the promoter-luciferase activity of p21, a p53 target gene, in SV-LECs (Figure 4B). Moreover, we performed a ChIP analysis to examine whether Sp1 or p53 is recruited to the endogenous *survivin* promoter region (−236 to −26) containing putative Sp1 and p53 binding sites, in WMJ-S-001-stimulated SV-LECs. As shown in Figure 4C, 6 h WMJ-S-001 exposure increased p53 binding to the *survivin* promoter region (−236/−26). This effect was accompanied by a concomitant decrease in Sp1 binding to the region.

p38-mediated signaling cascade has been shown previously to cause p53 phosphorylation and survivin reduction in cancer cells (23). We thus elucidated whether WMJ-S-001-induced p53 activation involves p38MAPK signaling in SV-LECs. As shown in Figure 4D, WMJ-S-001 time-dependently induced p38MAPK phosphorylation. A pharmacological p38MAPK inhibitor, p38 inhibitor III, significantly suppressed WMJ-S-001's effects in inducing p53 phosphorylation (Figure 4E) and survivin reduction (Figure 4F) in SV-LECs. It appears that WMJ-S-001 induces p38MAPK activation, resulting in p53 activation and survivin reduction in SV-LECs.

### WMJ-S-001 Suppressed Cell Proliferation, Invasion and Tube Formation of Primary Human LECs

**Figure 5:**
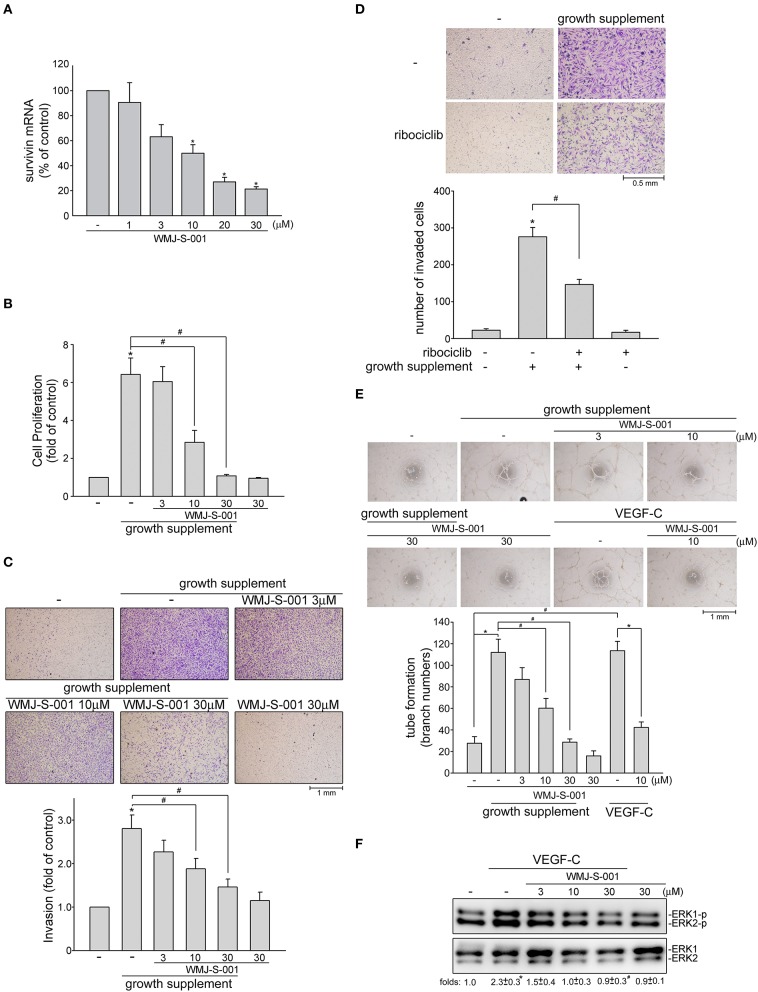
WMJ-S-001 caused survivin reduction and suppressed cell proliferation, invasion and tube formation in primary human LECs. **(A)** Primary human LECs were treated with WMJ-S-001 at indicated concentrations for 6 h. The extent of survivin mRNA was determined by Q-RT-PCR as described in the section Materials and Methods. Each column represents the mean ± S.E.M. of six independent experiments (*p < 0.05, compared with the control group). **(B)** Primary human LECs were starved in MV2 basal medium for 24 h. After starvation, cells were incubated in growth supplements-containing MV2 growth medium in the absence or presence of indicated concentrations of WMJ-S-001 for another 24 h. Cell proliferation was determined as described in the section Materials and Methods. Each column represents the mean ± SEM of ten independent experiments performed in duplicate (*p < 0.05, compared with the control group; #*p* < 0.05, compared with the control group in the presence of growth supplements). After starvation as described in **(B)**, cells were seeded in the top chamber in the absence or presence of indicated concentrations of WMJ-S-001 **(C)** or ribociclib (10 μM) **(D)** using growth supplements as chemo-attractant. After 24 h, invaded cells through the gelatin-coated membrane were stained and quantified. Each column represents the mean ± S.E.M. of six independent experiments. (*p < 0.05, compared with the control group; #*p* < 0.05, compared with the control group in the presence of growth supplements). **(E)** Primary human LECs were seeded on Matrigel in the presence of growth supplements or VEGF-C (50 ng/ml) with or without WMJ-S-001 at indicated concentrations. Cells were then photographed under phase-contrast after 16 h. Bar graphs show compiled data of average sprout arch numbers (*n* = 6) (*p < 0.05, compared with the control group; #*p* < 0.05, compared with the group treated with growth supplements or VEGF-C alone). **(F)** Cells were treated with WMJ-S-001 at indicated concentrations for 30 min followed by VEGF-C (50 ng/ml) exposure for another 20 min. The extent of ERK1/2 phosporylation was determined by immunoblotting. The complied results shown at the bottom of the blot represents the mean ± S.E.M. of three independent experiments (*p < 0.05, compared with the control group; #*p* < 0.05, compared with the group treated with WMJ-S-001 alone).

We next examined whether WMJ-S-001 reduces survivin expression and exhibits anti-lymphangiogenic activities in primary human LECs. As shown in Figure 5A, WMJ-S-001 at concentrations ranging from 10 to 30 μM significantly reduced survivin mRNA levels in human LECs (Figure 5A). WMJ-S-001's effects on cell proliferation in human LECs were examined using a BrdU incorporation assay. After starvation with MV2 basal medium for 24 h, human LECs were stimulated by MV2 growth medium in the absence or presence of WMJ-S-001 for another 24 h. The percentage of BrdU-labeled cells increased significantly after a 24 h treatment with MV2 growth medium. However, WMJ-S-001 reduced this increase in a concentration-dependent manner (Figure 5B). The effects of WMJ-S-001 on human LEC invasion were also determined using transwell invasion assay. As shown in Figure 5C, WMJ-S-001 significantly reduced the number of invading cells penetrating the gelatin-coated transwell filter barrier using MV2 growth medium as the chemoattractant (Figure 5C). We also examined whether ribociclib, a potent proliferation inhibitor targeting cyclin-dependent kinases (CDKs) 4/6 (42), affects human LEC invasion. As shown in Figure 5D, ribociclib, similar to WMJ-S-001, suppressed human LEC invasion. It is likely that WMJ-S-001's inhibitory effects on LEC invasion may also attribute to its anti-proliferative properties. Another key step of lymphangiogenesis is the tubular formation of LECs. We thus examined WMJ-S-001's effect on this step. Human LECs were seeded on the matrigel surface in complete MV2 growth medium in the presence of vehicle as control or WMJ-S-001. As shown in Figure 5E, cells incubated with MV2 growth medium became elongated, and formed capillary-like structure within 16 h. WMJ-S-001, however, concentration-dependently reduced the formation of capillary-like network (Figure 5E). Similarly, WMJ-S-001 also significantly reduced VEGF-C-induced tubular formation of human LECs (Figure 5E). Furthermore, WMJ-S-001 suppressed VEGF-C-induced ERK1/2 phosphorylation in human LECS (Figure 5F). Together these observations suggests that WMJ-S-001 exhibits anti-lymphangiogenic properties through suppressing cell proliferation, invasion and tubular formation of LECs. WMJ-S-001's actions in LECs may also attribute to survivin reduction.

## Discussion

Most cancer-related deaths are caused by tumor metastasis (2). Targeting the major routes for metastatic spread of tumor cells, angiogenesis and lymphangiogenesis, thus represents a promising therapeutic strategy for cancer intervention. To date, the European Medicines Agency (EMEA) or the U.S. Food and Drug Administration (FDA) has already approved several agents targeting angiogenesis including monoclonal antibodies and small molecule inhibitors for the treatment of certain types of cancer (43–45). In addition, some lymphangiogenesis inhibitors have been shown effective in reducing tumor progression and metastasis in solid tumors (46, 47). These observations led to increased efforts in discovering and developing novel agents targeting angiogenesis or lymphangiogenesis. Growing evidence shows beneficial effects of hydroxamate-based compounds in the treatment of cancer (24, 25, 31). Recently, we synthesized and identified a novel aliphatic hydroxamate-based compound, WMJ-S-001, that exhibits anti-tumor (22) and anti-angiogenesis (20) properties in *in vitro* and *in vivo* models. In this study, we further demonstrated that WMJ-S-001 also suppressed lymphangiogenesis using LECs as a cell model. We also demonstrated that p38MAPK-p53-survivin signaling might participate in WMJ-S-001's anti-lymphangiogenic actions.

Beyond its anti-apoptotic effects, survivin regulates a variety of cellular events such as cell cycle progression, cell migration and angiogenesis, which may enhance tumor metastasis and progression (16, 17, 37, 48). However, the association between endothelial survivin and lymphangiogenesis, remains to be fully defined. In the present study, we showed that endothelial survivin reduction significantly suppressed LEC invasion, a key step in lymphangiogenesis. Similar to previous studies (22, 40), we noted that WMJ-S-001-induced survivin reduction attributes to the activation of p53 in LECs. WMJ-S-001-activated p53 also led to cell cycle regulator p21 transcriptional activation, which blocks cell cycle machinery. It appears that WMJ-S-001's anti-proliferative effects in LECs may involve additional cell cycle regulatory proteins. In addition to suppressing cell cycle progression, WMJ-S-001 at concentrations higher than 10 μM also caused caspase 3 activation and apoptosis. It is likely that WMJ-S-001's anti-lymphangiogenic effects may also attribute to its apoptotic mechanisms when its concentrations is higher than 10 μM.

We showed that p38MAPK mediates p53 phosphorylation and survivin reduction in LECs after WMJ-S-001 exposure. The precise mechanisms underlying WMJ-S-001-induced p38MAPK activation in LECs remains to be established. Chen et al. (49) reported that activating Src homology 2 (SH2) domain-containing protein tyrosine phosphatase-1 (SHP-1)-PP2A-p38MAPK signaling cascade leads to p53 activation and vascular smooth muscle cell death. We have established that SHP-1 activation is involved in WMJ-S-001's anti-angiogenic effects in HUVECs (20). We also noted that SHP-1 signaling blockade by NSC-87877 reduces WMJ-S-001's effects on survivin and p21 levels in both HUVECs and HCT116 colorectal cancer cells (unpublished data). It raises the possibility that WMJ-S-001-induced p53 activation and subsequent cellular events may also involve SHP-1 in LECs.

VEGF-C activation of VEGFR-3 signaling plays a crucial role in lymphangiogenesis (7, 8). In addition to targeting p38MAPK-p53-survivin cascade, we noted that WMJ-S-001 suppresses VEGF-C-induced ERK phosphorylation and tube formation in primary human LECs. It suggests that inhibition of VEGF-C-VEGFR-3 signaling is causally related to WMJ-S-001's anti-lymphangiogenic effects. The inhibitory mechanisms of WMJ-S-001 in VEGF-C-stimulated LECs remain to be further investigated. The importance of protein tyrosine phosphatases (PTPs) in regulating VEGF effects has been highlighted recently in endothelial cells (50). Among these PTPs, density enhanced phosphatase (DEP)-1 (51), PTP1B (52), VE-PTP (53), and SHP-1(20) have been shown to negatively regulate VEGFR-2 signaling. In contrast, PTPs involved in the regulation of VEGFR-3 remains largely unknown. Deng et al. (54) recently showed that VE-PTP regulates VEGF-C-VEGFR-3 signaling in LECs. Together these findings suggest that PTPs may contribute to WMJ-S-001 inactivation of VEGF-C-VEGFR-3 signaling. It is worth to investigate whether certain PTP such as VE-PTP or SHP-1 contributes to WMJ-S-001's anti-lymphangiogenic actions.

Several lines of evidence demonstrated that angiogenesis inhibitors targeting the VEGF-VEGF-R signaling might enhance the evasive and adaptive resistance to the therapies in tumor cells. The underlying mechanisms by which the cancer cells develop resistance remain incompletely understood. The bevacizumab (a clinical used VEGF monoclonal antibody)-adapted tumor cells may switch their dependence to alternative proangiogenic signaling and enhancement of lymphatic-mediated metastasis (55). Sunitinib, a multi-targeted tyrosine kinase inhibitor, also induced evasive adaption in cancer cells through an alternative neurophilin 1(NRP-1)-mediated signaling (56). Whereas, sunitinib-adapted tumor exhibits less lymphatic metastasis, as it targets both lymphangiogenesis and angiogenesis (57). It is likely that single-target agents may be inadequate as therapeutics. We have shown in this study that p38MAPK-p53-mediated survivin reduction, at least in part, contributes to WMJ-S-001's anti-lymphangiogenic actions in LECs. Moreover, WMJ-S-001 has additional properties such as anti-tumor (22), anti-angiogenic (20) and anti-inflammatory (28) activities. The precise mechanisms underlying these activities remain to be fully define. Together these findings, however, support WMJ-S-001's potential as a promising lead compound in developing novel agents for oncologic therapy.

## Data Availability Statement

The datasets generated for this study are available on request to the corresponding author.

## Author Contributions

Designed the experiments: S-WH, H-YY, W-JH, S-WW, Y-FH, and M-JH. Performed the experiments: S-WH, W-CC, M-CY, Y-FH, and M-JH. Analyzed the data: S-WH, H-YY, W-CC, M-CY, Y-FH, and M-JH. Contributed reagents, synthesized, and WMJ-J compounds: W-JH and S-WW. Wrote the paper: S-WH, H-YY, Y-FH, and M-JH.

### Conflict of Interest

The authors declare that the research was conducted in the absence of any commercial or financial relationships that could be construed as a potential conflict of interest.

## Abbreviations

IAP: inhibitor of apoptosis protein
LEC: lymphatic endothelial cell
MTT, 3-(4,5-dimethylthiazol-2-yl)-2: 5-diphenyl tetrazolium bromide
p38MAPK: p38 mitogen-activated protein kinase
PI: propidium iodide
VEGF: vascular endothelial growth factor.
